# Examination of the temporal sequence between social media use and well-being in a representative sample of adults

**DOI:** 10.1007/s00127-022-02363-2

**Published:** 2022-09-19

**Authors:** Hannah K. Jarman, Siân A. McLean, Susan J. Paxton, Chris G. Sibley, Mathew D. Marques

**Affiliations:** 1 School of Psychology and Public Health, La Trobe University, Melbourne, Australia; 2 School of Psychology, The University of Auckland, Auckland, New Zealand

**Keywords:** Social media, Well-being, Psychological distress, Life satisfaction, Prospective

## Abstract

Given insufficient prospective evidence for relationships between social media use and well-being among adults, the present study examined the temporal sequence between social media use and psychological distress and life satisfaction, and explored age and gender differences. A representative sample of adults (*N* = 7331; 62.4% women; *M*_age_ = 51.94; *SD* = 13.48; 15–94 years) were surveyed annually across four waves. Cross-lagged panel models demonstrated bidirectional relationships between social media use and well-being. Higher psychological distress and lower life satisfaction predicted higher social media use more strongly than the reverse direction, with effects particularly pronounced for the impact of psychological distress. Although the patterns of findings were relatively consistent across age and gender, results suggested that women and middle- and older-aged adults experience detrimental effects of social media use on well-being, which may drive subsequent increased use of social media. The bidirectional relationships suggest that adults who experience psychological distress or lower life satisfaction may seek to use social media as a way to alleviate poor well-being. However, paradoxically, this maladaptive coping mechanism appears to drive increased social media use which in turn can exacerbate poor well-being. Clinicians should be aware of these bidirectional relationships and work with clients towards replacing ineffective strategies with more helpful coping approaches. As this study used a simplistic measure of social media use, future research should address this limitation and explore nuanced relationships afforded by assessing specific social media activities or exposure to certain types of content.

**Supplementary Information:**

The online version contains supplementary material available at 10.1007/s00127-022-02363-2.

## Introduction

Subjective well-being refers to an individual’s appraisal and evaluation of their own life [1], with both cognitive and affective components. Subjective well-being (referred to hereafter as well-being), thus, may be reflected in several ways including both negative (psychological distress) and positive (life satisfaction) aspects, the focus of the present research. Evidence suggests that greater well-being predicts greater health, longevity, and psychological functioning (e.g., [1, 2]) as well as lower mental illness [3]. Well-being is also associated with lower risk of mortality [4]. As a result, well-being has been identified as an important global health issue [5, 6]. Consequently, to promote health and wellness, it is valuable to explore factors which may contribute to well-being.

One factor which has received recent attention is the potential role of social media use in relation to well-being. Over the past decade, social media use has risen dramatically, with the proportion of adults who use at least one social media platform growing from 37% in 2009 to 72% in 2019 [7]. Given that social media has become an important part of daily life, researchers have proposed that high levels of social media use may impact well-being [8]. Negative consequences for well-being are proposed to result from upward social comparisons made with social media content which largely presents idealised and positive images and life-circumstances of others, fuelling envy and discontent with one’s own life [8, 9]. Also, consistent with the displacement hypothesis, time spent online might displace other crucial activities which support well-being, including sleep, physical activity, and face-to-face social contact (e.g., [10]), resulting in diminished well-being. Alternatively, social media use, especially use involving direct exchanges with others, may improve well-being by contributing to social capital and greater connectedness with friends [8, 9, 11].

Empirical findings regarding relationships between social media use and well-being are largely cross-sectional (e.g., [12–14]), restricting understanding of temporal sequencing. Overall, meta-analyses have demonstrated a small, significant relationship between time spent on social networking sites and psychological well-being (*r* = − 0.07; [15, 16]), although it appears that this relationship is somewhat dependent on indicators of well-being. While the relationship between time spent on social media and poor well-being (e.g., depressive symptoms) is small, the relationship with positive well-being (e.g., life satisfaction) is non-significant or close to zero [15, 17].

Few longitudinal studies have examined whether social media use predicts well-being outcomes over time, with most focusing on adolescents and youth. Furthermore, these provide inconsistent information about temporal sequencing. While some research has found that higher time spent on social media predicts poor well-being, particularly among girls [18], others have either described the effect as negligible or found no such effect [19, 20]. Experimental research among young adults demonstrated that greater Facebook use predicted declines in life satisfaction over a 2-week period [21]. Notably, this study was conducted before the major upsurge in social media use on platforms such as Instagram, and had a brief follow-up period. Conversely, a six-wave longitudinal study over 3 years found no evidence to suggest that social media use impacts life satisfaction [22]. Further investigation is needed to clarify these inconsistencies.

It is also possible that poorer well-being may contribute to greater social media use. Uses and gratification theory [23] posit that humans are purposive in their media consumption whereby they seek to fulfil certain psychological needs. Thus, it could be that well-being drives social media use. For example, high psychological distress may prompt increased use to provide distraction from daily stressors and/or a way to disengage from life [24]. Additionally, social media may be used to a greater extent to cope with negative feelings and attempt to enhance well-being. In line with this, a mediation analysis of cross-sectional data found that among internet users (aged 14–39 years), psychosocial well-being (loneliness, depression, and anxiety) positively predicted social media use and this effect was mediated by social comparison tendency and fear of missing out [25]. Consequently, it is possible that the relationship between social media use and well-being is bidirectional.

Although plausible, the potential for a bidirectional relationship between social media use and indicators of well-being has largely not been examined in longitudinal designs, particularly among adults. Bidirectional relationships have been found among adolescent girls, whereby greater life satisfaction predicted slightly lower social media use while greater social media use predicted decreases in life satisfaction over time [20]. Further, adolescent girls with higher depressive symptoms reported greater social media use over time, whereas no evidence demonstrated that social media use predicted depressive symptoms for male or female adolescents and young adults [26].

The lack of empirical research examining the prospective relationship between social media use and well-being among adults is of concern. Adults across the age spectrum are now regular social media users. For example, estimates in the United States indicate that 90% of adults of 18–29 years, 82% of adults of 30–49 years, 69% of adults of 50–64 years, and 40% of adults over 65 years report using at least one social media platform [7]. Consequently, the need to understand relationships between social media use and well-being in adults is essential, with some evidence suggesting that the relationship between social media use and psychological distress is larger among adults than adolescents [14]. If a negative impact of social media use is observed among adults, interventions need to be considered to mitigate such effects, including updating public health messaging and policy development within the social media landscape.

It is also important to examine these relationships across age groups within adulthood, and in men compared to women. Younger adults are more likely to spend greater time engaging with photos and using appearance-focused platforms Instagram and Snapchat, than older adults [27, 28]. Greater appearance-focused social media use could promote greater social comparisons and concerns about presentation of the ideal self and thus could lead to poorer well-being in younger than older adults. Similarly, in developed countries, women are more likely to use social media than men [29], and thus, for a similar reason, may be more vulnerable to subsequent poorer well-being. Relatedly, empirical research suggests that women are more likely to engage in upward social comparisons than men [30], which may undermine well-being [8].

### The present study

Research generally supports a relationship between high social media use and poor well-being. However, given the lack of prospective evidence, particularly among adults, the primary aim of the present study was to examine the temporal relationships between social media use and psychological distress and life satisfaction among a representative sample of adults. Furthermore, limited research has examined these relationships in a large sample of adults across a wide age range. Therefore, the secondary aims of this study were to examine if these relationships differed by age group (younger, middle-aged, and older-aged adults) and by gender (men, women).

## Method

### Participants and procedure

The New Zealand Attitudes and Values Study (NZAVS) is a longitudinal study of personal characteristics and health outcomes using a national probability sample of New Zealand adults. Data reported here were sampled in the 2016 (T1), 2017 (T2), 2018 (T3), and 2019 (T4) waves; the waves for which the social media and well-being measures were implemented. Participants responded to a mail-out survey or completed the survey online. Sample, response rate, and retention information for these four waves are reported in Supplementary Table 1. See [31] for further details about the NZAVS sampling procedure and related information.

Participants retained across all four timepoints in our sample (*N* = 7331; 62.4% women, 37.6% men) were aged between 15 and 94 years (*M* = 51.94, *SD* = 13.48). The University of Auckland Human Participants Ethics Committee approved all procedures, and participants gave informed consent.

### Materials

#### Demographic information

Participants provided information on age, gender, and ethnicity, among other variables, as part of an omnibus survey. A more detailed description of ethnicity across the waves can be found in Supplementary Table 1. Socio-economic status (SES) was determined by the New Zealand Socio-Economic Index [32]. This measure is based on occupational status where participant’s open-ended responses are classified according to the Australian and New Zealand Standard Classification of Occupations (ANZSCO) Level 3 (10 = low, 90 = high).

#### Social media use

Participants estimated the number of hours spent on social media use (e.g., Facebook) in the previous week. Self-reported media exposure is moderately reliable and highly stable [33], and single-item measures of self-reported time spent on social media have demonstrated good convergent validity with other measures, such as frequency [34] and duration of social media use [35].

#### Psychological distress

Participants responded to the K6 psychological distress scale [36] to indicate how often they felt distressed from 0 (*none of the time)* to 4 (*all of the time*) during the last 30 days. Sample items are “feel hopeless?” and “feel restless or fidgety?”. Item responses were averaged to create a scale score. The K6 is strongly associated with present and prospective mood and anxiety disorders, and has satisfactory reliability [36–38]. From the total score, a standard cut-off of ≥ 13 (scaled average ≥ 2.617) is used to identify individuals with a diagnosable mental illness severe enough to cause functional limitations which may require treatment [39, 40]. Reliability coefficients in the present sample were acceptable (α = 0.85 for each time-point; see Table 1).

**Table 1:** Summary of intercorrelations, means, and standard deviations for cross-lagged panel model for times 1, 2, 3, and 4 of psychological distress and social media use

	*M*	*(SD)*	*α*	*N*	1	2	3	4	5	6	7	8	9	10	11
1. Social Media Use T1	3.366	(6.415)	‒	13,576	1										
2. Social Media Use T2	4.190	(7.689)	‒	21,188	0.581	1									
3. Social Media Use T3	3.964	(7.025)	‒	16,663	0.613	0.639	1								
4. Social Media Use T4	4.288	(7.736)	‒	46,413	0.564	0.554	0.625	1							
5. Psychological Distress T1	0.826	(0.648)	.848	13,904	0.159	0.159	0.155	0.163	1						
6. Psychological Distress T2	0.878	(0.679)	.850	21,849	0.165	0.177	0.174	0.165	0.734	1					
7. Psychological Distress T3	0.842	(0.654)	.850	17,031	0.166	0.178	0.190	0.169	0.709	0.738	1				
8. Psychological Distress T4	0.900	(0.691)	.852	47,462	0.165	0.174	0.183	0.169	0.699	0.722	0.757	1			
9. Gender T1	.373	(.484)	‒	13,892	− 0.123	− 0.118	− 0.129	− 0.125	− 0.053	− 0.051	− 0.055	− 0.053	1		
10. Age T1	50.799	(13.906)	‒	13,942	− 0.257	− 0.254	− 0.261	− 0.246	− 0.257	− 0.260	− 0.271	− 0.271	0.121	1	
11. Ethnicity T1	.897	(.303)	‒	13,942	− 0.097	− 0.101	− 0.106	− 0.104	− 0.064	− 0.065	− 0.063	− 0.079	− 0.018	0.088	1
12. SES T1	54.362	(16.039)	‒	13,813	− 0.028	− 0.023	− 0.015	− 0.038	− 0.108	− 0.090	− 0.082	− 0.095	− 0.067	− 0.064	0.054

*n* = 55,218

Ethnicity is coded as 1 = NZ Euro, 0 = other. SES = Socio-Economic Status. Gender is coded as 0 = Women, 1 = Men

All zero-order correlations are significant at p < 0.01

#### Life satisfaction

Participants reported their level of agreement with two statements from the Satisfaction With Life scale [41] using a seven-point scale (1 = *strongly disagree*; 7 = *strongly agree)*. Responses to items: (a) “I am satisfied with my life”, and (b) “In most ways my life is close to ideal”, were averaged to create a scale score. Spearman's rank correlation coefficients indicated acceptable internal consistency (*ρ* = 0.61–0.67) and are reported in Table 2. This short version of the scale has been used extensively in previous research, demonstrating convergent and discriminant validity [42, 43].

**Table 2:** Summary of intercorrelations, means, and standard deviations for cross-lagged panel model for times 1, 2, 3, and 4 of life satisfaction and social media use

	*M*	*(SD)*	*ρ*	*N*	1	2	3	4	5	6	7	8	9	10	11
1. Social Media Use T1	3.366	(6.415)	‒	13,576	1										
2. Social Media Use T2	4.190	(7.689)	‒	21,188	0.580	1									
3. Social Media Use T3	3.964	(7.025)	‒	16,663	0.613	0.638	1								
4. Social Media Use T4	4.288	(7.736)	‒	46,413	0.563	0.554	0.625	1							
5. Life Satisfaction T1	5.231	(1.184)	.657	13,536	− 0.090	− 0.080	− 0.077	− 0.077	1						
6. Life Satisfaction T2	5.224	(1.195)	.648	21,292	− 0.091	− 0.090	− 0.081	− 0.078	0.748	1					
7. Life Satisfaction T3	5.264	(1.210)	.666	16,459	− 0.089	− 0.079	− 0.066	− 0.065	0.721	0.753	1				
8. Life Satisfaction T4	5.305	(1.206)	.608	47,635	− 0.083	− 0.090	− 0.080	− 0.082	0.695	0.716	0.753	1			
9. Gender T1	0.373	(.484)	‒	13,892	− 0.122	− 0.117	− 0.128	− 0.123	− 0.049	− 0.045	− 0.035	− 0.063	1		
10. Age T1	50.799	(13.906)	‒	13,942	− 0.254	− 0.249	− 0.258	− 0.242	0.078	0.089	0.086	0.111	0.120	1	
11. Ethnicity T1	0.897	(0.303)	‒	13,942	− 0.096	− 0.099	− 0.105	− 0.103	0.042	0.053	0.039	0.050	− 0.018	0.086	1
12. SES T1	54.362	(16.039)	‒	13,813	− 0.026	− 0.020	− 0.014	− 0.037	0.120	0.104	0.095	0.108	− 0.068	− 0.066	0.053

*Notes. n* = 55,175

Ethnicity is coded as 1 = NZ Euro, 0 = other. SES = Socio-Economic Status. Gender is coded as 0 = Women, 1 = Men

All zero-order correlations are significant at *p* < 0.01

### Data analysis

To investigate our aims, we specified a series of stationary cross-lagged panel models (CLPM) using Mplus version 8.0 [44] to assess the effects of social media use_T-1_ (Time 1, 2, 3) on well-being indicators_T_ (Time 2, 3, 4) and vice-versa—well-being indicators_*T *− 1_ on social media use_*T*_. Models were specified separately for each well-being indicator (psychological distress and life satisfaction), and unstandardized estimates reported. We constrained the auto-regressive and cross-lagged associations to be the same at each measurement point, because we were more interested in the overall and comparative effects of well-being and social media use on each other than whether these effects change over time. To model these associations as a stationary process, all congeneric paths were constrained to equality (e.g., the auto-regressive association between social media use at Time 1 and social media use at Time 2 was constrained to be equal to the same auto-regressive association at Time 2 and Time 3 and so on). We also covaried all initial predictors with each other and on each auto-regressive and cross-lagged path. We used a threshold of *p* < 0.01 to indicate significance of findings and describe the magnitude of effects using Cohen’s *d* [45].

All models were run with maximum-likelihood estimation with robust standard errors [46], and we used full information maximum-likelihood (FIML) to impute missing data [47]. Bias-corrected (BC) 99% confidence intervals (CIs) were estimated using 1000 bootstrapped resamples (with replacement). Our predictions controlled for gender, age, SES, and ethnicity.

To explore whether associations between well-being indicators and social media use varied across age and gender, we performed additional multigroup models grouped by age (< 35 years; ≥ 35 < 50 years; ≥ 50 years), and by gender (men; women). We examined whether the strengths in the bidirectional relationships between well-being and social media use were different across these pre-defined categories. All syntax is available on the NZAVS website.

In each model, auto-regressive relationships between outcome variables (e.g., well-being indicator_*T* − 1_ to well-being indicator_*T*_) were examined, and, as typically observed, there was high stability across time, with these auto-regressive relationships being significant and large in all models. As these were not the focus of the present research, details of these relationships are provided in Supplementary Materials. Descriptive statistics and zero-order correlations between variables for each analysis are also provided in the manuscript tables and in Supplementary Materials.

### Pre-registration

We pre-registered our aims, method, and data analysis strategy including example syntax (http://tiny.cc/wbsm).

## Results

### Descriptive characteristics

Tables 1 and 2 present descriptive statistics and correlations between variables across all time points for psychological distress and life satisfaction, respectively. Social media hours ($r$ = 0.56, *p* < 0.001), psychological distress ($r$ = 0.70, *p* < 0.001), and life satisfaction ($r$ = 0.55, *p* < 0.001) showed high levels of rank-order stability over 4 years (i.e., between the annual assessments at the first and last time-point). Transformations to Fisher *z*-scores, and subsequent transformations back to Pearson correlation coefficients, indicated that the average wave-to-wave correlations for social media use ($\overline{r }$ = 0.62, *p* < 0.001), psychological distress ($\overline{r }$ = 0.74, *p* < 0.001), and life satisfaction ($\overline{r }$ = 0.75, *p* < 0.001) were also high. These data show that social media use and indicators of well-being were stable over time. Average year-to-year intercorrelations between psychological distress and life satisfaction were also moderate ($\overline{r }$ = − 0.47, *p* < 0.001).

On average, the sample reported around 3–4 of social media use, low levels of psychological distress, and levels of life satisfaction above the mid-point of the scale. Across the four waves, the proportion of participants reporting levels of psychological distress categorised as severe ranged from 4.90 to 6.67% (≥ 13 cut-off), which is similar to other large samples (∼ 4%; [48, 49]). There were small positive zero-order correlations between social media use and psychological distress, and small negative correlations between social media use and life satisfaction. Being younger, having lower SES, and being from a non-New Zealand European background were associated with higher social media use and psychological distress, as well as lower life satisfaction. Women were significantly more likely than men to report higher social media use and levels of psychological distress, but also higher life satisfaction.

### Cross-lagged paths between psychological distress and social media use over time

**Fig. 1:**
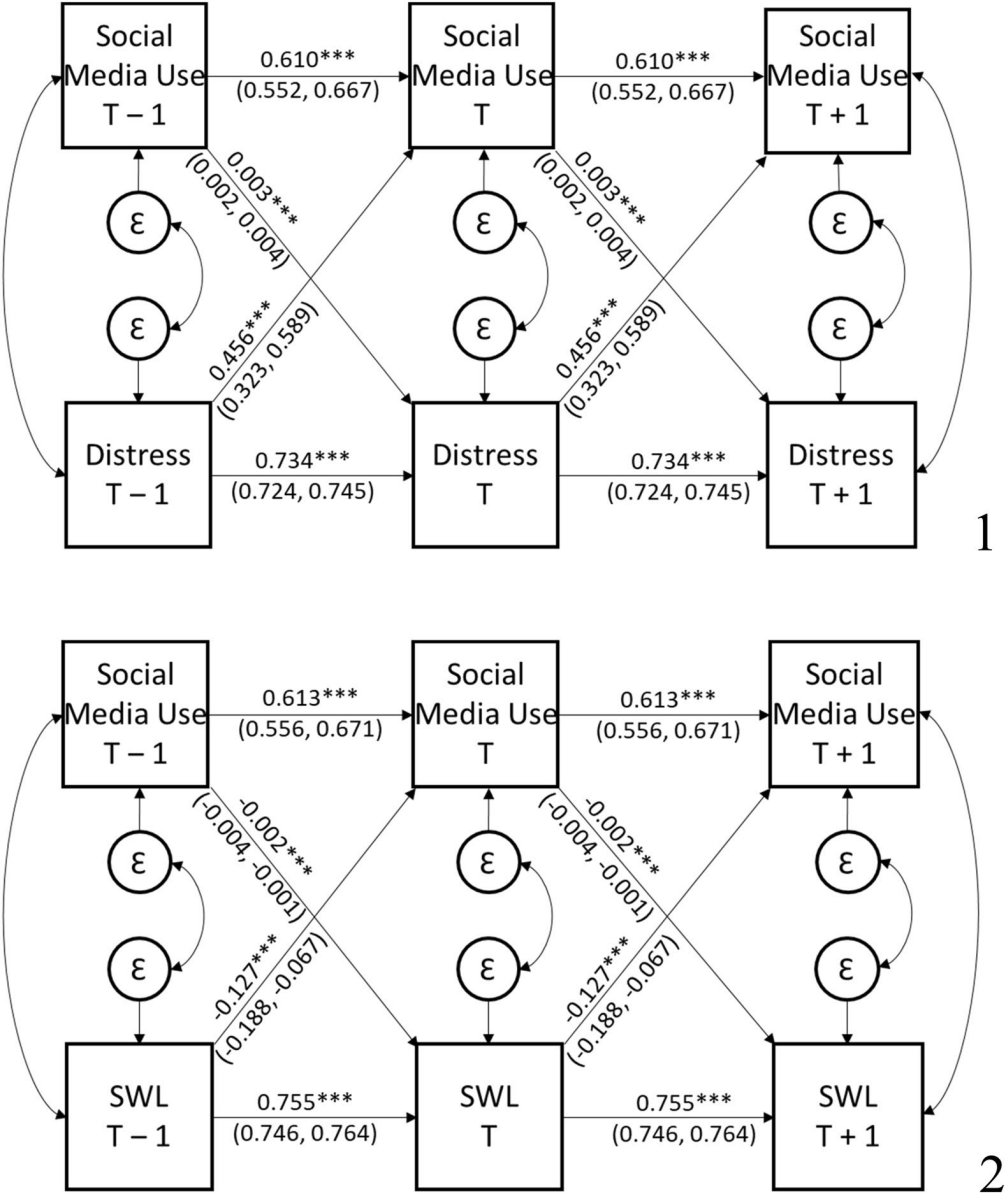
Stationary cross-lagged panel model of the associations between social media use and psychological distress (1) and life satisfaction (2). Distress = Psychological Distress, SWL = Life Satisfaction. Coefficients are unstandardized (with bias corrected 99% confidence intervals). ***p < 0.001

The cross-lagged model of social media use and psychological distress is presented in Fig. 1 (all estimates including covariates are presented in Supplementary Table 2). Given our large sample size, obtaining a non-significant Chi-square would be very unlikely, *χ*^2^(22) = 1033.184, *p* < 0.001, comparative fit index (*CFI*) = 0.924. Indices less influenced by complexity suggested adequate fit of the model to the observed covariance matrix; root-mean-square error of approximation (*RMSEA*) = 0.029, and standardized root-mean-square residual (*SRMR*) = 0.056 [i.e., acceptable fit represented by RMSEA ≤ 0.08 and SRMR ≤ 0.05; 50]. There was a significant positive association from social media use_*T *− 1_ to psychological distress_*T*_ (*B* = 0.003, *p* < 0.001, BC CI_99_ [0.002, 0.004]) and from psychological distress_*T *− 1_ to social media use_*T*_ (*B* = 0.456, *p* < 0.001, BC CI_99_ [0.323, 0.589]). The association from psychological distress_*T *− 1_ to social media use_*T*_ was significantly stronger than the association from social media use_*T *− 1_ to psychological distress_*T*_, *χ*^2^(1) = 76.748, *p* < 0.001. Results suggested that a one-unit increase in psychological distress was associated with an average increase 1 year later of 27 min and 22 s in social media use per day, and a 1-h increase in social media use was associated with a 0.003 unit increase in psychological distress 1 year later.

### Cross-lagged paths between life satisfaction and social media use over time

The cross-lagged model of social media use and life satisfaction is presented in Fig. 1 (all estimates including covariates are presented in Supplementary Table 3). The specified model was an adequate fit with the data, *χ*^2^(22) = 1005.465, *p* < 0.001, *CFI* = 0.925, *RMSEA* = 0.028, *SRMR* = 0.056. There was a significant and small negative association from social media use_*T *− 1_ to life satisfaction_T_ (*B* = − 0.002, *p* < 0.001, BC CI_99_ [− 0.004, − 0.001]) and from life satisfaction_*T *− 1_ to social media use_*T*_ (*B* = -0.127, *p* < 0.001, BC CI_99_ [− 0.188, − 0.067]). The association from life satisfaction_*T *− 1_ to social media use_T_ was significantly stronger than the association from social media use_*T *− 1_ to life satisfaction_T_, *χ*^2^(1) = 28.508, *p* < 0.001. Results suggested that a one-unit increase in life satisfaction was associated with an average decrease 1 year later of 7 min and 37 s in social media use per day. A 1-h increase in social media use was associated with a 0.002 unit decrease in life satisfaction 1 year later.

### Multigroup analyses

#### Age

**Fig. 2:**
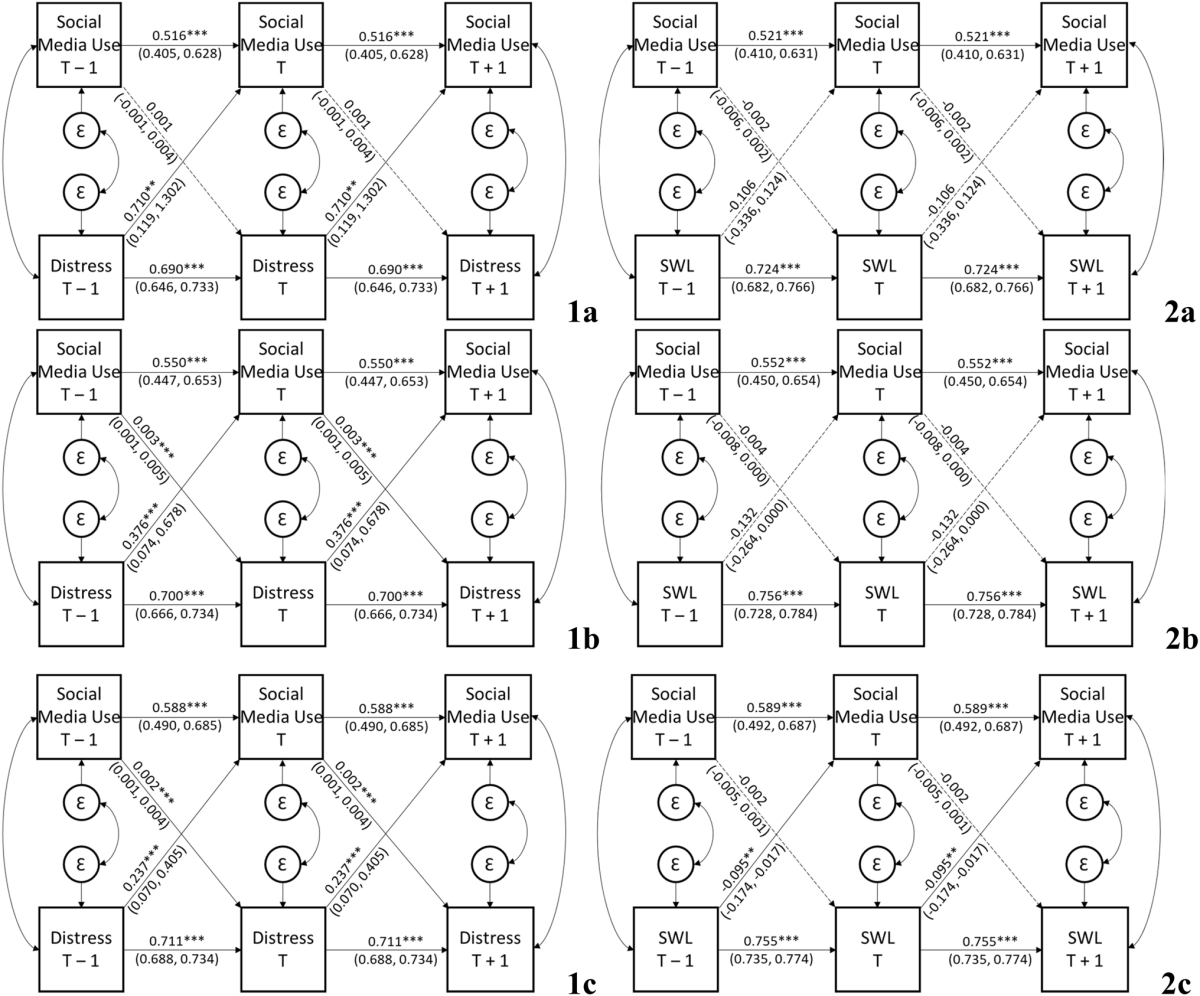
Stationary cross-lagged panel model of the associations between social media use and psychological distress (1) and life satisfaction (2) in young (a) and middle (b), and older-aged (c) adults. SWL = Life Satisfaction. Coefficients are unstandardized (with bias corrected 99% confidence intervals). Dashed lines reflect non-significant paths. **p < 0.01, ***p < 0.001

**Fig. 3:**
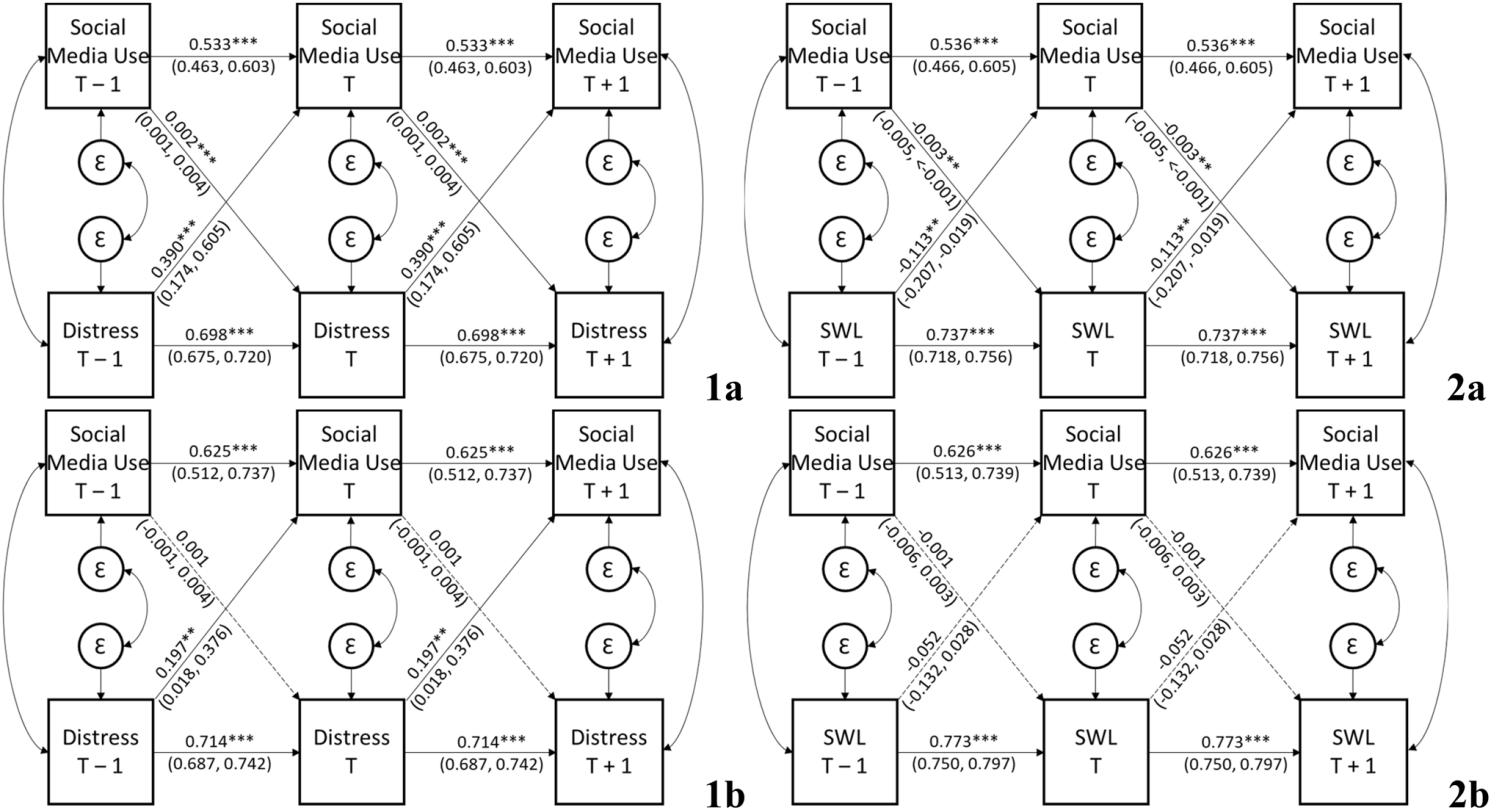
Stationary cross-lagged panel model of the associations between social media use and psychological distress (1) and life satisfaction (2) in women (a) and men (b). SWL = Life Satisfaction. Coefficients are unstandardized (with bias corrected 99% confidence intervals). Dashed lines reflect non-significant paths. **p < 0.01, ***p < 0.001

Multigroup analyses for age and gender are presented in Figs. 2 and 3, respectively, and in Supplementary Materials (Tables 4, 5, 6 and 7). In the multigroup age model comparisons, the model fit were acceptable for both models; psychological distress *χ*^2^(81) = 1489.174, *p* < 0.001, *CFI* = 0.829, *RMSEA* = 0.061 [CI_90_ = 0.059,0.064], *SRMR* = 0.067, and life satisfaction *χ*^2^(81) = 1430.364, *p* < 0.001, *CFI* = 0.850, *RMSEA* = 0.060 [CI_90_ = 0.057,0.063], *SRMR* = 0.064.

For younger individuals (< 35; *M*_age_ = 27.67 [*SD* = 4.17], *n* = 1943), there was a significant association between psychological distress_*T *− 1_ and social media use_*T*_ (*B* = 0.710, *p* = 0.002, BC CI_99_ [0.119,1.302]), which was stronger than the non-significant association between social media use_*T *− 1_ and psychological distress_*T*_ (*B* = 0.001, *p* = 0.177, BC CI_99_ [− 0.001,0.004]), *χ*^2^(1) = 9.536, *p* = 0.002. For life satisfaction, the associations between life satisfaction_*T *− 1_ and social media use_*T*_ (*B* = − 0.106, *p* = 0.236, BC CI_99_ [− 0.336,0.124]) and between social media use_*T *− 1_ and life satisfaction_T_ (*B* = − 0.002, *p* = 0.149, BC CI_99_ [− 0.006,0.002]) were both statistically non-significant.

For middle-aged individuals (≥ 35 < 50; *M*_age_ = 42.87 [*SD* = 4.24], *n* = 4079), the significant association between psychological distress_T-1_ and social media use_T_ (*B* = 0.376, *p* = 0.001, BC CI_99_ [0.074,0.678) was stronger than the significant association between social media use_*T *− 1_ and psychological distress_*T*_ (*B* = 0.003, *p* = 0.001, BC CI_99_ [0.001,0.005]), *χ*^2^(1) = 10.173, *p* = 0.001. In contrast, neither of the associations from life satisfaction_T-1_ to social media use_*T*_ (*B* = − 0.132, *p* = 0.010, BC CI_99_ [− 0.264, < 0.001]) or from social media use_*T *− 1_ to life satisfaction_T_ (*B* = − 0.004, *p* = 0.016, BC CI_99_ [− 0.008, < 0.001]) reached statistical significance.

For older-aged individuals (> 50; *M*_age_ = 60.65 [*SD* = 8.08], *n* = 7870), the significant association between psychological distress_T-1_ and social media use_T_ (*B* = 0.237, *p* < 0.001, BC CI_99_ [0.070,0.405) was stronger than the significant association of social media use_T-1_ with psychological distress_T_ (*B* = 0.002, *p* < 0.001, BC CI_99_ [0.001,0.004]), *χ*^2^(1) = 13.152, *p* < 0.001. Similarly, for life satisfaction in older-aged individuals, the life satisfaction_*T *− 1_ and social media use_T_ association was statistically significant (*B* = − 0.095, *p* = 0.002, BC CI_99_ [− 0.174,− 0.017]), and was stronger than the non-significant association between social media use_*T *− 1_ and life satisfaction_T_ (*B* = − 0.002, *p* = 0.088, BC CI_99_ [− 0.005,0.001]), *χ*^2^(1) = 9.517, *p* = 0.002.

As a follow-up, we tested whether the associations from well-being indicators to social media use were significantly different across age groups. For psychological distress, there was no significant difference between any age group comparisons: younger and middle-aged individuals (*χ*^2^[1] = 1.680, *p* = 0.195), middle-aged and older individuals (*χ*^2^[1] = 1.069, *p* = 0.301), and younger and older-aged individuals (*χ*^2^[1] = 3.923, *p* = 0.048). Similarly, for life satisfaction, there was no significant difference between any age group comparisons: younger and middle-aged individuals (*χ*^2^[1] = 0.064, *p* = 0.800), middle-aged and older individuals (*χ*^2^[1] = 0.378, *p* = 0.539), and younger and older-aged individuals (*χ*^2^[1] = 0.012, *p* = 0.911).

#### Gender

Finally, we examined the strength of relationships between well-being indicators and social media use grouped by gender. The model fit was acceptable for both psychological distress *χ*^2^(50) = 1154.398, *p* < 0.001, *CFI* = 0.859, *RMSEA* = 0.056 [CI_90_ = 0.053,0.059], *SRMR* = 0.061 and life satisfaction, *χ*^2^(50) = 1142.998, *p* < 0.001, *CFI* = 0.869, *RMSEA* = 0.056 [CI_90_ = 0.053,0.059], *SRMR* = 0.061. As shown in Fig. 3, the cross-lagged paths suggested a different pattern of results by gender and well-being indicator.

For women, the significant association between psychological distress_T-1_ and social media use_T_ (*B* = 0.390, *p* < 0.001, BC CI_99_ [0.174,0.605]) was significantly stronger than the significant association between social media use_T-1_ and psychological distress_T_ (*B* = 0.002, *p* < 0.001, BC CI_99_ [0.001,0.004), *χ*^2^(1) = 21.501, *p* < 0.001. Similarly, the significant association between life satisfaction_*T *− 1_ and social media use_T_ (*B* = − 0.113, *p* = 0.002, BC CI_99_ [− 0.207, − 0.019]) was significantly stronger than the significant association between social media use_*T *− 1_ and life satisfaction_T_ (*B* = − 0.003, *p* = 0.004, BC CI_99_ [− 0.005, < 0.001]), *χ*^2^(1) = 9.329, *p* = 0.002.

For men, the significant association between psychological distress_T-1_ to social media use_T_ (*B* = 0.197, *p* = 0.005, BC CI_99_ [0.018,0.376]) was stronger than the non-significant association between social media use_*T *− 1_ and psychological distress_*T*_ (*B* = 0.001, *p* = 0.152, BC CI_99_ [− 0.001,0.004), *χ*^2^(1) = 7.972, *p* = 0.005. In contrast, neither the association between life satisfaction_*T *− 1_ to social media use_*T*_ (*B* = − 0.052, *p* = 0.096, BC CI_99_ [− 0.132,0.028])_,_ or the association between social media use_*T *− 1_ and life satisfaction_*T*_ (*B* = − 0.001, *p* = 0.502, BC CI_99_ [− 0.006,0.003]) reached statistical significance.

Given the consistently larger associations from well-being indicators to social media use in women compared to men, we tested whether these were significantly different across gender. Results suggested that neither the association from psychological distress to social media use (*χ*^2^[1] = 3.141, *p* = 0.076), nor the association from life satisfaction to social media use, was significantly different across gender (*χ*^2^[1] = 1.616, *p* = 0.204).

## Discussion

This study aimed to examine the temporal relationships between social media use and psychological distress and life satisfaction among a representative sample of adults, and to explore age and gender differences in these relationships. Although a bidirectional relationship was found, higher psychological distress and lower life satisfaction predicted higher social media use more strongly than the reverse direction. Patterns of findings were relatively consistent across age and gender groups. Overall, effects were small, except for the relationship between psychological distress and social media use, whereby a one-unit increase in psychological distress was associated with an average increase in social media use of almost 30 min per day 1 year later in the total sample.

The bidirectional relationship between social media use and well-being is consistent with some (e.g., [20]), but not all (e.g., [26]), research among adolescents. However, our study is the first to demonstrate this bidirectional effect among adults. These findings support uses and gratification theory [23]; in that, a stronger relationship was found for psychological needs, namely distress and life satisfaction, influencing social media use than the reverse relationship. It is possible that adults turn to social media if they are experiencing negative emotions, including using it as a tool to distract or avoid dealing with emotions [51]. Alternatively, users may see social media as a way to help cope with problems and serve their needs, including feedback seeking. However, greater social media use was not associated with improved well-being over time, so if this is the case, it would not appear to be a successful strategy. It is possible that social media use instead encourages comparisons with idealised presentations and promotes fear of missing out, resulting in diminished well-being [8, 25]. In line with displacement hypothesis, this effect may be especially powerful if face-to-face social support is replaced with social media use.

Relationships between well-being and social media use were dependent on the measure of well-being used. Psychological distress, a negative indicator, was more closely associated with social media use than was life satisfaction, a positive indicator. This finding is consistent with previous meta-analyses [15, 17], highlighting the importance of examining varied indicators of well-being to understand these nuanced relationships. Psychological distress, an affective component of well-being, may be more responsive to the immediate environment and subject to day-to-day fluctuations, whereas life satisfaction, an indicator of cognitive well-being, may encompass a more stable self-concept [52].

When effects were examined by age group, relationships from psychological distress and life satisfaction to social media use remained stronger than the reverse for all groups. Interestingly, life satisfaction predicted social media use only in the older-age group. Across age groups, the only significant associations from social media use to well-being indicators were for the middle- and older-age groups where the well-being indicator was psychological distress. This is particularly interesting given that the majority of research has focused on younger populations, typically finding that engagement with a specific social media platform, such as Facebook, predicts lower life satisfaction over short time periods (e.g., [21]). As younger adults are prolific users of social media and considered digital natives, their day-to-day use of social media may have less impact on general well-being. These novel findings of bidirectional relationships among middle- and older-aged adults indicate the need for further research to replicate this effect and to examine how and why people in these age groups use social media.

For both men and women, the association from psychological distress to social media use was significantly stronger than the reverse. This finding was mirrored for life satisfaction but only for women. Furthermore, our results indicate that higher social media use predicted poorer well-being only among women. The relationship from social media to poorer well-being is consistent with research among adolescents (e.g., [18]). It is also consistent with research related to another area of distress, body dissatisfaction, where in earlier analyses of the present sample, social media use predicted body dissatisfaction among women [53]. This gender-specific effect may be due to women being more likely than men to use social media to seek feedback and to engage in comparisons, which may contribute to negative self-evaluations and depressive symptoms [54, 55]. Despite moderate social media use, adult men have been found to engage less in comparisons, or even find comparisons inspiring if they are deemed achievable [30], which may explain our findings.

The present study has several implications. The findings provide preliminary support for uses and gratification theory among adults within a social media context [23], whereby poorer well-being predicted higher social media use 1 year later. Although this direction was found to be stronger than the reverse, a bidirectional relationship was observed. Consequently, it is possible that these relationships may work as a continuous loop whereby attempts to alleviate poor well-being by engaging with social media are maladaptive and the increased social media use in turn exacerbates poor well-being. Future research is required to explore this. Practically, our findings suggest that adults may turn to social media as a coping strategy for poor well-being. Accordingly, clinicians, especially those working with women and middle or older-aged adults, should work with clients experiencing distress to explore why and how they use social media, and help support them to build alternative positive coping strategies. For example, using social media to escape life or comparing against others could be replaced with positive strategies such as problem solving or seeking social support, which promotes well-being [11]. Furthermore, public health campaigns may be used on social media which target individuals who appear to be experiencing psychological distress, including individuals who post key terms or follow certain types of content (e.g., [56]). If well-being can be promoted within the community, individuals may be less likely to turn to social media for purposes of distress alleviation.

Although the present study has several strengths including the inclusion of a large, representative sample of adults over time, limitations must be considered. The single-item measure of duration of social media use was brief, so could not capture the richness of user’s experience. Nuanced measures of specific social media activities or content may provide a more in-depth understanding of how participants engage with social media so should be used in future research. In addition, assessments in the present study were 1 year apart. Assessments at closer timepoints (i.e., ecological momentary assessments) may capture different fluctuations in the relationships between social media use and well-being. The present study focused on well-being. While the K6 cut offs indicate that approximately 5–6% of participants were experiencing severe psychological distress, future research should also consider the prevalence of psychiatric disorders among samples as well as the possibility of confounding effects. Finally, although representative of adults in New Zealand, these findings should be replicated elsewhere to confirm their generalisability.

## Conclusions

The present study indicates that although the relationships between social media use and well-being are bidirectional, the effect of well-being on social media use is stronger than the reverse. These effects were more pronounced for psychological distress than life satisfaction. Furthermore, these findings suggest that women and middle- and older-aged adults experience detrimental effects of social media use on well-being, which may drive subsequent increases in social media use. The consistency of effects across genders and the age range suggests that attempts to redirect from social media, or modify the type of social media use that people turn to when distressed, are likely to be equally applicable across the adult lifespan.

## Supplementary Information

Below is the link to the electronic supplementary material.

Supplementary file1 (DOCX 86 KB)
