# Supplementary material for: Examination of the temporal sequence between social media use and well-being in a representative sample of adults
Source: Soc Psychiatry Psychiatr Epidemiol. 2022 Sep 19;58(8):1247–58. doi: 10.1007/s00127-022-02363-2 (PMC10366027; doi:10.1007/s00127-022-02363-2)
Supplement: Supplementary file 1 — Supplementary file1 (DOCX 86 KB) [file 127_2022_2363_MOESM1_ESM.docx]

# Supplementary Materials

**Supplementary Table 1**

Sample, Response Rate, and Retention Information for Four Annual Waves of the New Zealand Attitudes and Values Survey.

|  | T7 (2015) | T8 (2016) | T9 (2017) | T10 (2018) |
| --- | --- | --- | --- | --- |
| Sample | 13,942 | 21,936 | 17,072 | 47,951 |
| Response Rate (1 or more previous wave) | 13,941 | 13,781 | 16,931 | 18,010 |
| Unmatched / unsolicited opt-ins | 1 | 488 | 141 | ‒ |
| Booster Samples | ‒ | 7,667 | ‒ | 29,193 |
| Retention T-1 | 12,550 | 11,933 | 15,784 | 14,049 |
| Retention Rate T-1 | 79.3% | 85.6% | 72.0% | 82.3% |
| Retention Rate over 5 years | 51.3% | 51.4% | 42.5% | ‒ |
| Ethnicity^a^ |  |  |  |  |
| European | 12,506 | 19,478 | 15,606 | 42,495 |
| Māori | 1,672 | 2,743 | 2,007 | 4,691 |
| Pacific Nations peoples | 427 | 574 | 466 | 1,037 |
| Asian | 545 | 1,001 | 700 | 2,538 |

*Notes.* Information from: Sibley, C. G. (Updated 20 January 2020) Sampling procedure and sample details for the New Zealand Attitudes and Values Study. *NZAVS Technical Documents, e01*. ^a^ Participants could identify with multiple ethnic groups, therefore their responses could be counted multiple times.

**Supplementary Table 2**

Path Coefficients of the Associations Between Social Media Use and Psychological Distress.

| Predictors  *T-1* | Outcome  *T* |  |  |  |  | Covariates T1^#^ |  |  |  |  |
| --- | --- | --- | --- | --- | --- | --- | --- | --- | --- | --- |
|  | Social Media Use |  | Psychological Distress | |  | Social Media Use | Psychological Distress | Ethnicity | SES | Gender |
|  | *B (SE)* | *99% CI* | *B (SE)* | *99% CI* |  |  |  |  |  |  |
| Social Media Use | 0.610 (0.022)* | (0.552,0.667) | 0.003 (<0.001)* | (0.002,0.004) |  | 1 | 0.043 (0.025) | ‒ | ‒ | ‒ |
| Psychological Distress | 0.456 (0.052)* | (0.323,0.589) | 0.734 (0.004)* | (0.724,0.745) |  | 0.703* (0.054) | 1 | ‒ | ‒ | ‒ |
| Ethnicity | -1.039 (0.423) | (-2.129,-0.051) | -0.067 (0.024)* | (-0.129,-0.005) |  | -0.195* (0.027) | -0.014* (0.002) | 1 | ‒ | ‒ |
| SES | -0.019 (0.005)* | (-0.031,-0.006) | -0.002 (<0.001)* | (-0.003,-0.001) |  | -3.483* (1.046) | -1.235* (0.102) | 0.278* (0.045) | 1 | ‒ |
| Gender | -0.664 (0.103)* | (-0.930,-0.399) | -0.008 (0.010) | (-0.033,0.017) |  | -0.383* (0.024) | -0.017* (0.003) | -0.003 (0.001) | -0.518* (0.067) | 1 |
| Age | -0.043 (0.006)* | (-0.057,-0.028) | -0.004 (<0.001)* | (-0.004,-0.003) |  | -22.921* (0.965) | -2.323* (0.077) | 0.374* (0.036) | -14.079* (1.838) | 0.813* (0.054) |

*Notes. n* = 55,218.
Ethnicity is coded as 1 = NZ Euro, 0 = other. SES = Socio Economic Status. Gender is coded as 0 = Women, 1 = Men.
^#^ modelled as covariances. Covariance above the diagonal is T4 covariance.
**p* < .01

**Supplementary Table 3**

Path Coefficients of the Associations Between Social Media Use and Life Satisfaction.

| Predictors T-1 | Outcome *T* |  |  |  |  | Covariates T1^#^ |  |  |  |  |
| --- | --- | --- | --- | --- | --- | --- | --- | --- | --- | --- |
|  | Social Media |  | Life Satisfaction | |  | Social Media Use | Life Satisfaction | Ethnicity | SES | Gender |
|  | *B (SE)* | *99% CI* | *B (SE)* | *99% CI* |  |  |  |  |  |  |
| Social Media | 0.613 (0.022)* | (0.556,0.671) | -0.002 (0.001)* | (-0.004,-0.001) |  | 1 | -0.073 (0.039) | ‒ | ‒ | ‒ |
| Life Satisfaction | -0.127 (0.023)* | (-0.188,-0.067) | 0.755 (0.004)* | (0.746,0.764) |  | -0.719* (0.086) | 1 | ‒ | ‒ | ‒ |
| Ethnicity | -1.066 (0.429) | (-2.170,0.038) | 0.075 (0.041) | (-0.029,0.180) |  | -0.190* (0.027) | 0.016* (0.003) | 1 | ‒ | ‒ |
| SES | -0.019 (0.005)* | (-0.032,-0.006) | 0.003 (0.001)* | (0.001,0.005) |  | -3.095* (1.036) | 2.356* (0.173) | 0.266* (0.045) | 1 | ‒ |
| Gender | -0.680 (0.101)* | (-0.942,-0.419) | -0.100 (0.017)* | (-0.144,-0.056) |  | -0.379* (0.024) | -0.028* (0.005) | -0.003 (0.001) | -0.529* (0.067) | 1 |
| Age | -0.047 (0.006)* | (-0.062,-0.032) | 0.004 (0.001)* | (0.003,0.006) |  | -22.505* (0.961) | 1.248* (0.137) | 0.365* (0.036) | -14.695* (1.826) | 0.800* (0.056) |

*Notes. n* = 55,218.
Ethnicity is coded as 1 = NZ Euro, 0 = other. SES = Socio Economic Status. Gender is coded as 0 = Women, 1 = Men.
^#^ modelled as covariances. Covariance above the diagonal is T4 covariance.
**p* < .01

**Gender multigroup**

**Psychological Distress**

**Supplementary Table 4a**

Summary of Intercorrelations, Means, and Standard Deviations for Cross Lagged Panel Model for Times 1, 2, 3, and 4 of Psychological Distress and Social Media Use by Gender

|  | Women  *n* = 8,712 | |  |  |  |  |  |  |  |  |  |  |  |  | Men *n* = 5,180 | |  |
| --- | --- | --- | --- | --- | --- | --- | --- | --- | --- | --- | --- | --- | --- | --- | --- | --- | --- |
|  | *M* | *(SD)* | *N* | 1 | 2 | 3 | 4 | 5 | 6 | 7 | 8 | 9 | 10 | 11 | *M* | *(SD)* | *N* |
| 1. Social Media Use T1 | 3.941 | (7.044) | 8,472 | 1 | .654 | .677 | .463 | .130 | .120 | .107 | .101 | -.236 | -.117 | -.020 | 2.396 | (5.060) | 5,056 |
| 2. Social Media Use T2 | 4.268 | (7.561) | 7,242 | .484 | 1 | .621 | .462 | .119 | .116 | .116 | .107 | -.232 | -.107 | -.006 | 2.606 | (5.706) | 4,347 |
| 3. Social Media Use T3 | 4.216 | (7.026) | 6,152 | .514 | .620 | 1 | .649 | .120 | .122 | .122 | .115 | -.213 | -.121 | -.011 | 2.504 | (4.962) | 3,650 |
| 4. Social Media Use T4 | 4.067 | (7.144) | 6,353 | .493 | .491 | .526 | 1 | .110 | .104 | .103 | .122 | -.186 | -.097 | -.017 | 2.414 | (5.132) | 3,807 |
| 5. Psychological Distress T1 | 0.848 | (0.660) | 8,689 | .129 | .121 | .129 | .138 | 1 | .736 | .703 | .683 | -.224 | -.052 | -.102 | 0.786 | (0.624) | 5,165 |
| 6. Psychological Distress T2 | 0.853 | (0.662) | 7,430 | .142 | .151 | .160 | .144 | .703 | 1 | .740 | .713 | -.21 | -.054 | -.076 | 0.792 | (0.640) | 4,419 |
| 7. Psychological Distress T3 | 0.829 | (0.653) | 6,285 | .138 | .137 | .171 | .134 | .677 | .706 | 1 | .738 | -.239 | -.078 | -.074 | 0.759 | (0.614) | 3,703 |
| 8. Psychological Distress T4 | 0.827 | (0.647) | 6,501 | .136 | .128 | .158 | .132 | .670 | .697 | .734 | 1 | -.228 | -.076 | -.084 | 0.769 | (0.619) | 3,865 |
| 9. Age T1 | 49.545 | (13.672) | 8,712 | -.222 | -.217 | -.238 | -.225 | -.240 | -.250 | -.247 | -.251 | 1 | .082 | -.067 | 52.961 | (13.996) | 5,180 |
| 10. Ethnicity T1 | .902 | (.457) | 1,943 | -.073 | -.078 | -.078 | -.075 | -.062 | -.061 | -.040 | -.063 | .088 | 1 | .053 | .889 | (.315) | 5,180 |
| 11. SES T1 | 55.239 | (15.659) | 8,624 | -.033 | -.027 | -.017 | -.044 | -.107 | -.092 | -.079 | -.093 | -.061 | .050 | 1 | 52.934 | (16.561) | 5,140 |

*Notes. N* = 13,892.
Women’s covariances below the diagonal, men’s covariances above the diagonal. Ethnicity is coded as 1 = NZ Euro, 0 = other. SES = Socio Economic Status.
Women *p* < .01 where *r* ≥ .028. Men *p* < .01 where *r* ≥ .036.

**Supplementary Table 4b**

Path Coefficients of the Associations Between Social Media Use and Psychological Distress for Women.

| Predictors T-1 | Outcome *T* |  |  |  |  | Covariates T1^#^ |  |  |
| --- | --- | --- | --- | --- | --- | --- | --- | --- |
|  | Social Media |  | Psychological Distress | |  | Social Media Use | Psychological Distress | SES |
|  | *B (SE)* | *99% CI* | *B (SE)* | *99% CI* |  |  |  |  |
| Social Media | 0.533 (0.027)* | (0.463,0.603) | 0.002 (0.001)* | (0.001,0.004) |  | 1 | 0.034 (0.036) | ‒ |
| Psychological Distress | 0.390 (0.084)* | (0.174,0.605) | 0.698 (0.009)* | (0.675,0.720) |  | 0.601* (0.065) | 1 | ‒ |
| Ethnicity | -0.524 (0.374) | (-1.487,0.439) | -0.052 (0.025) | (-0.117,0.013) |  | ‒ | ‒ | ‒ |
| SES | -0.017 (0.006)* | (-0.032,-0.003) | -0.002 (<0.001)* | (-0.003,-0.001) |  | -3.740* (1.218) | -1.108* (0.119) | 1 |
| Age | -0.047 (0.007)* | (-0.064,-0.030) | -0.004 (<0.001)* | (-0.005,-0.003) |  | -21.347* (1.190) | -2.164* (0.100) | -12.933* (2.233) |

*Notes. n* = 8,712.
Ethnicity is coded as 1 = NZ Euro, 0 = other. SES = Socio Economic Status.
^#^ modelled as covariances. Covariance above the diagonal is T4 covariance.
**p* < .01

**Supplementary Table 4c**

Path Coefficients of the Associations Between Social Media Use and Psychological Distress for Men.

| Predictors T-1 | Outcome *T* |  |  |  |  | Covariates T1^#^ |  |  |
| --- | --- | --- | --- | --- | --- | --- | --- | --- |
|  | Social Media |  | Psychological Distress | |  | Social Media Use | Psychological Distress | SES |
|  | *B (SE)* | *99% CI* | *B (SE)* | *99% CI* |  |  |  |  |
| Social Media | 0.625 (0.044)* | (0.512,0.737) | 0.001 (0.001) | (-0.001,0.004) |  | 1 | 0.080* (0.030) | ‒ |
| Psychological Distress | 0.197 (0.069)* | (0.018,0.376) | 0.714 (0.011)* | (0.687,0.742) |  | 0.408* (0.075) | 1 | ‒ |
| Ethnicity | -0.255 (0.334) | (-1.115,0.604) | -0.034 (0.029) | (-0.108,0.040) |  | ‒ | ‒ | ‒ |
| SES | -0.004 (0.004) | (-0.015,0.006) | -0.001 (<0.001)* | (-0.002,<0.001) |  | -1.719 (1.377) | -1.048* (0.150) | 1 |
| Age | -0.020 (0.007)* | (-0.039,-0.001) | -0.003 (0.001)* | (-0.004,-0.001) |  | -16.726* (1.319) | -1.958* (0.123) | -15.432* (3.056) |

*Notes. n* = 5,180.
Ethnicity is coded as 1 = NZ Euro, 0 = other. SES = Socio Economic Status.
^#^ modelled as covariances. Covariance above the diagonal is T4 covariance.
**p* < .01

**Life Satisfaction**

**Supplementary Table 5a**

Summary of Intercorrelations, Means, and Standard Deviations for Cross Lagged Panel Model for Times 1, 2, 3, and 4 of Life Satisfaction and Social Media Use by Gender

|  | Women  *n* = 8,712 | |  |  |  |  |  |  |  |  |  |  |  |  | Men *n* = 5,180 | |  |
| --- | --- | --- | --- | --- | --- | --- | --- | --- | --- | --- | --- | --- | --- | --- | --- | --- | --- |
|  | *M* | *(SD)* | *N* | 1 | 2 | 3 | 4 | 5 | 6 | 7 | 8 | 9 | 10 | 11 | *M* | *(SD)* | *N* |
| 1. Social Media Use T1 | 3.941 | (7.044) | 8,472 | 1 | .654 | .677 | .464 | -.102 | -.098 | -.091 | -.094 | -.236 | -.116 | -.018 | 2.396 | (5.060) | 5,056 |
| 2. Social Media Use T2 | 4.268 | (7.561) | 7,242 | .484 | 1 | .622 | .462 | -.100 | -.104 | -.084 | -.115 | -.232 | -.106 | -.005 | 2.606 | (5.706) | 4,347 |
| 3. Social Media Use T3 | 4.216 | (7.026) | 6,152 | .514 | .620 | 1 | .649 | -.093 | -.086 | -.090 | -.086 | -.213 | -.121 | -.010 | 2.504 | (4.962) | 3,650 |
| 4. Social Media Use T4 | 4.067 | (7.144) | 6,353 | .493 | .491 | .526 | 1 | -.072 | -.067 | -.055 | -.065 | -.187 | -.097 | -.017 | 2.414 | (5.132) | 3,807 |
| 5. Life Satisfaction T1 | 5.276 | (1.192) | 8,478 | -.078 | -.053 | -.062 | -.071 | 1 | .765 | .742 | .707 | .115 | .018 | .1 | 5.157 | (1.165) | 5,009 |
| 6. Life Satisfaction T2 | 5.291 | (1.189) | 7,272 | -.077 | -.060 | -.066 | -.081 | .729 | 1 | .784 | .740 | .12 | .036 | .09 | 5.187 | (1.180) | 4,364 |
| 7. Life Satisfaction T3 | 5.307 | (1.202) | 6,121 | -.085 | -.062 | -.059 | -.062 | .700 | .733 | 1 | .760 | .128 | .032 | .084 | 5.230 | (1.197) | 3,591 |
| 8. Life Satisfaction T4 | 5.397 | (1.164) | 6,491 | -.067 | -.056 | -.071 | -.071 | .676 | .695 | .740 | 1 | .146 | .029 | .092 | 5.239 | (1.167) | 3,859 |
| 9. Age T1 | 49.545 | (13.672) | 8,712 | -.222 | -.217 | -.238 | -.225 | .055 | .068 | .060 | .090 | 1 | .082 | -.066 | 52.961 | (13.996) | 5,180 |
| 10. Ethnicity T1 | .902 | (.298) | 8,712 | -.073 | -.078 | -.078 | -.075 | .051 | .058 | .040 | .057 | .088 | 1 | .052 | .889 | (.315) | 5,180 |
| 11. SES T1 | 55.239 | (15.659) | 8,624 | -.033 | -.026 | -.016 | -.043 | .122 | .101 | .095 | .106 | -.060 | .049 | 1 | 52.934 | (16.561) | 5,140 |

*Notes. N* = 13,892.
Women’s covariances below the diagonal, men’s covariances above the diagonal.
Ethnicity is coded as 1 = NZ Euro, 0 = other. SES = Socio Economic Status.
Women *p* < .01 where *r* ≥ .028. Men *p* < .01 where *r* ≥ .036.

**Supplementary Table 5b**

Path Coefficients of the Associations Between Social Media Use and Life Satisfaction for Women.

| Predictors T-1 | Outcome *T* |  |  |  |  | Covariates T1^#^ |  |  |
| --- | --- | --- | --- | --- | --- | --- | --- | --- |
|  | Social Media |  | Life Satisfaction | |  | Social Media Use | Life Satisfaction | SES |
|  | *B (SE)* | *99% CI* | *B (SE)* | *99% CI* |  |  |  |  |
| Social Media | 0.536 (0.027)* | (0.466,0.605) | -0.003 (0.001)* | (-0.005,<0.001) |  | 1 | -0.043 (0.056) | ‒ |
| Life Satisfaction | -0.113 (0.036)* | (-0.207,-0.019) | 0.737 (0.007)* | (0.718,0.756) |  | -0.645* (0.121) | 1 | ‒ |
| Ethnicity | -0.513 (0.373) | (-1.474,0.448) | 0.084 (0.041) | (-0.023,0.191) |  | ‒ | ‒ | ‒ |
| SES | -0.018 (0.006)* | (-0.032,-0.004) | 0.003 (0.001)* | (0.001,0.004) |  | -3.748* (1.218) | 2.265* (0.207) | 1 |
| Age | -0.051 (0.007)* | (-0.068,-0.034) | 0.004 (0.001)* | (0.002,0.006) |  | -21.363* (1.190) | 0.883* (0.173) | -12.924* (2.233) |

*Notes. n* = 8,712.
Ethnicity is coded as 1 = NZ Euro, 0 = other. SES = Socio Economic Status.
^#^ modelled as covariances. Covariance above the diagonal is T4 covariance.
**p* < .01

**Supplementary Table 5c**

Path Coefficients of the Associations Between Social Media Use and Life Satisfaction for Men.

| Predictors T-1 | Outcome *T* |  |  |  |  | Covariates T1^#^ |  |  |
| --- | --- | --- | --- | --- | --- | --- | --- | --- |
|  | Social Media |  | Life Satisfaction | |  | Social Media Use | Life Satisfaction | SES |
|  | *B (SE)* | *99% CI* | *B (SE)* | *99% CI* |  |  |  |  |
| Social Media | 0.626 (0.044)* | (0.513,0.739) | -0.001 (0.002) | (-0.006,0.003) |  | 1 | -0.031 (0.058) | ‒ |
| Life Satisfaction | -0.052 (0.031) | (-0.132,0.028) | 0.773 (0.009)* | (0.750,0.797) |  | -0.606* (0.101) | 1 | ‒ |
| Ethnicity | -0.267 (0.335) | (-1.130,0.597) | -0.008 (0.058) | (-0.157,0.142) |  | ‒ | ‒ | ‒ |
| SES | -0.005 (0.004) | (-0.015,0.006) | 0.002 (0.001) | (<0.001,0.004) |  | -1.573 (1.367) | 1.945* (0.284) | 1 |
| Age | -0.022 (0.007)* | (-0.041,-0.003) | 0.004 (0.001)* | (0.001,0.006) |  | -16.732* (1.318) | 1.860* (0.229) | -15.360* (3.055) |

*Notes. n* = 5,180.
Ethnicity is coded as 1 = NZ Euro, 0 = other. SES = Socio Economic Status.
^#^ modelled as covariances. Covariance above the diagonal is T4 covariance.
**p* < .01

**Age multigroup**

**Psychological Distress**

**Supplementary Table 6a**

Summary of Intercorrelations, Means, and Standard Deviations for Cross Lagged Panel Model for Times 1, 2, 3, and 4 of Psychological Distress and Social Media Use for Younger Adults

|  | *M* | *(SD)* | *N* | 1 | 2 | 3 | 4 | 5 | 6 | 7 | 8 | 9 | 10 |
| --- | --- | --- | --- | --- | --- | --- | --- | --- | --- | --- | --- | --- | --- |
| 1. Social Media Use T1 | 6.504 | (9.730) | 1,922 | 1 |  |  |  |  |  |  |  |  |  |
| 2. Social Media Use T2 | 7.263 | (11.184) | 1,487 | .482 | 1 |  |  |  |  |  |  |  |  |
| 3. Social Media Use T3 | 7.031 | (10.109) | 1,229 | .518 | .562 | 1 |  |  |  |  |  |  |  |
| 4. Social Media Use T4 | 6.650 | (9.474) | 1,258 | .462 | .411 | .554 | 1 |  |  |  |  |  |  |
| 5. Psychological Distress T1 | 1.137 | (0.684) | 1,938 | .111 | .070 | .086 | .139 | 1 |  |  |  |  |  |
| 6. Psychological Distress T2 | 1.169 | (0.695) | 1,517 | .139 | .123 | .160 | .163 | .646 | 1 |  |  |  |  |
| 7. Psychological Distress T3 | 1.152 | (0.711) | 1,239 | .095 | .094 | .175 | .141 | .631 | .690 | 1 |  |  |  |
| 8. Psychological Distress T4 | 1.146 | (0.677) | 1,272 | .065 | .028 | .119 | .121 | .597 | .632 | .702 | 1 |  |  |
| 9. Gender T1 | .299 | (.457) | 1,943 | -.078 | -.064 | -.118 | -.137 | -.046 | -.075 | -.051 | -.04 | 1 |  |
| 10. Ethnicity T1 | .853 | (.354) | 1,943 | -.060 | -.054 | -.001 | -.047 | -.020 | -.027 | -.013 | .017 | -.032 | 1 |
| 11. SES T1 | 54.817 | (16.651) | 1,927 | -.081 | -.047 | -.053 | -.090 | -.166 | -.137 | -.142 | -.156 | -.008 | .010 |

*Notes. n* = 1,943.
Ethnicity is coded as 1 = NZ Euro, 0 = other. SES = Socio Economic Status. Gender is coded as 0 = Women, 1 = Men.
*p* < .01 where *r* ≥ .059

**Supplementary Table 6b**

Path Coefficients of the Associations Between Social Media Use and Psychological Distress for Younger Adults.

| Predictors T-1 | Outcome *T* |  |  |  |  | Covariates T1^#^ |  |
| --- | --- | --- | --- | --- | --- | --- | --- |
|  | Social Media |  | Psychological Distress | |  | Social Media Use | Psychological Distress |
|  | *B (SE)* | *99% CI* | *B (SE)* | *99% CI* |  |  |  |
| Social Media | 0.516 (0.043)* | (0.405,0.628) | 0.001 (0.001) | (-0.001,0.004) |  | 1 | 0.138 (0.101) |
| Psychological Distress | 0.710 (0.230)* | (0.119,1.302) | 0.690 (0.017)* | (0.646,0.733) |  | 0.733* (0.200) | 1 |
| Ethnicity | -1.242 (0.759) | (-3.198,0.713) | 0.048 (0.046) | (-0.070,0.165) |  | ‒ | ‒ |
| SES | -0.032 (0.017) | (-0.075,0.012) | -0.002 (0.001)* | (-0.005,<0.001) |  | -13.187 (3.813) | -1.901* (0.280) |
| Gender | -1.384 (0.390)* | (-2.389,-0.380) | -0.001 (0.031) | (-0.080,0.079) |  | ‒ | ‒ |

*Notes. n* = 1,943.
Ethnicity is coded as 1 = NZ Euro, 0 = other. SES = Socio Economic Status. Gender is coded as 0 = Women, 1 = Men.
^#^ modelled as covariances. Covariance above the diagonal is T4 covariance.
**p* < .01

**Supplementary Table 6c**

Summary of Intercorrelations, Means, and Standard Deviations for Cross Lagged Panel Model for Times 1, 2, 3, and 4 of Psychological Distress and Social Media Use for Mid Aged Adults

|  | *M* | *(SD)* | *N* | 1 | 2 | 3 | 4 | 5 | 6 | 7 | 8 | 9 | 10 |
| --- | --- | --- | --- | --- | --- | --- | --- | --- | --- | --- | --- | --- | --- |
| 1. Social Media Use T1 | 3.765 | (6.303) | 3,997 | 1 |  |  |  |  |  |  |  |  |  |
| 2. Social Media Use T2 | 4.057 | (6.443) | 3,303 | .477 | 1 |  |  |  |  |  |  |  |  |
| 3. Social Media Use T3 | 3.979 | (6.314) | 2,743 | .504 | .666 | 1 |  |  |  |  |  |  |  |
| 4. Social Media Use T4 | 3.911 | (6.510) | 2,912 | .480 | .522 | .461 | 1 |  |  |  |  |  |  |
| 5. Psychological Distress T1 | 0.886 | (0.637) | 4,074 | .094 | .100 | .097 | .092 | 1 |  |  |  |  |  |
| 6. Psychological Distress T2 | 0.893 | (0.636) | 3,340 | .090 | .093 | .104 | .087 | .702 | 1 |  |  |  |  |
| 7. Psychological Distress T3 | 0.875 | (0.631) | 2,777 | .119 | .109 | .130 | .071 | .659 | .674 | 1 |  |  |  |
| 8. Psychological Distress T4 | 0.883 | (0.641) | 2,960 | .120 | .108 | .113 | .092 | .637 | .671 | .712 | 1 |  |  |
| 9. Gender T1 | .329 | (.470) | 4,079 | -.113 | -.106 | -.117 | -.099 | -.018 | -.012 | -.003 | -.014 | 1 |  |
| 10. Ethnicity T1 | .888 | (.315) | 4,079 | -.086 | -.101 | -.114 | -.095 | -.038 | -.029 | -.029 | -.069 | -.006 | 1 |
| 11. SES T1 | 56.204 | (16.044) | 4,037 | -.036 | -.043 | -.034 | -.051 | -.123 | -.11 | -.085 | -.110 | -.069 | .060 |

*Notes. n* = 4,079.
Ethnicity is coded as 1 = NZ Euro, 0 = other. SES = Socio Economic Status. Gender is coded as 0 = Women, 1 = Men.
*p* < .01 where *r* ≥ .041.

**Supplementary Table 6d**

Path Coefficients of the Associations Between Social Media Use and Psychological Distress for Mid Aged Adults.

| Predictors T-1 | Outcome *T* |  |  |  |  | Covariates T1^#^ |  |
| --- | --- | --- | --- | --- | --- | --- | --- |
|  | Social Media |  | Psychological Distress | |  | Social Media Use | Psychological Distress |
|  | *B (SE)* | *99% CI* | *B (SE)* | *99% CI* |  |  |  |
| Social Media | 0.550 (0.040)* | (0.447,0.653) | 0.003 (0.001)* | (0.001,0.005) |  | 1 | 0.110 (0.057) |
| Psychological Distress | 0.376 (0.117)* | (0.074,0.678) | 0.700 (0.013)* | (0.666,0.734) |  | 0.379* (0.091) | 1 |
| Ethnicity | -0.705 (0.586) | (-2.214,0.805) | -0.093 (0.036) | (-0.187,>0.001) |  | ‒ | ‒ |
| SES | -0.012 (0.008) | (-0.032,0.007) | -0.002 (0.001)* | (-0.004,-0.001) |  | -3.599 (1.770) | -1.258* (0.169) |
| Gender | -0.589 (0.205)* | (-1.116,-0.061) | -0.023 (0.018) | (-0.070,0.025) |  | ‒ | ‒ |

*Notes. n* = 4,079.
Ethnicity is coded as 1 = NZ Euro, 0 = other. SES = Socio Economic Status. Gender is coded as 0 = Women, 1 = Men.
^#^ modelled as covariances. Covariance above the diagonal is T4 covariance.
**p* < .01

**Supplementary Table 6e**

Summary of Intercorrelations, Means, and Standard Deviations for Cross Lagged Panel Model for Times 1, 2, 3, and 4 of Psychological Distress and Social Media Use for Older Aged Adults

|  | *M* | *(SD)* | *N* | 1 | 2 | 3 | 4 | 5 | 6 | 7 | 8 | 9 | 10 |
| --- | --- | --- | --- | --- | --- | --- | --- | --- | --- | --- | --- | --- | --- |
| 1. Social Media Use T1 | 2.359 | (4.989) | 7,609 | 1 |  |  |  |  |  |  |  |  |  |
| 2. Social Media Use T2 | 2.653 | (5.600) | 6,799 | .546 | 1 |  |  |  |  |  |  |  |  |
| 3. Social Media Use T3 | 2.662 | (4.992) | 5,830 | .549 | .596 | 1 |  |  |  |  |  |  |  |
| 4. Social Media Use T4 | 2.550 | (5.425) | 5,990 | .456 | .454 | .585 | 1 |  |  |  |  |  |  |
| 5. Psychological Distress T1 | 0.716 | (0.613) | 7,842 | .074 | .073 | .076 | .069 | 1 |  |  |  |  |  |
| 6. Psychological Distress T2 | 0.726 | (0.625) | 6,992 | .077 | .094 | .083 | .063 | .712 | 1 |  |  |  |  |
| 7. Psychological Distress T3 | 0.697 | (0.596) | 5,972 | .060 | .075 | .075 | .066 | .685 | .719 | 1 |  |  |  |
| 8. Psychological Distress T4 | 0.697 | (0.595) | 6,134 | .068 | .089 | .090 | .070 | .684 | .706 | .727 | 1 |  |  |
| 9. Gender T1 | .414 | (.493) | 7,869 | -.109 | -.115 | -.111 | -.105 | -.025 | -.017 | -.030 | -.023 | 1 |  |
| 10. Ethnicity T1 | .912 | (.283) | 7,869 | -.065 | -.064 | -.083 | -.055 | -.054 | -.057 | -.052 | -.063 | -.037 | 1 |
| 11. SES T1 | 53.325 | (15.793) | 7,800 | -.004 | .006 | .015 | -.006 | -.097 | -.078 | -.074 | -.081 | -.074 | .070 |

*Notes. n* = 7,869.
Ethnicity is coded as 1 = NZ Euro, 0 = other. SES = Socio Economic Status. Gender is coded as 0 = Women, 1 = Men.
*p* < .01 where *r* ≥ .030.

**Supplementary Table 6f**

Path Coefficients of the Associations Between Social Media Use and Psychological Distress for Older Aged Adults.

| Predictors T-1 | Outcome *T* |  |  |  |  | Covariates T1^#^ |  |
| --- | --- | --- | --- | --- | --- | --- | --- |
|  | Social Media |  | Psychological Distress | |  | Social Media Use | Psychological Distress |
|  | *B (SE)* | *99% CI* | *B (SE)* | *99% CI* |  |  |  |
| Social Media | 0.588 (0.038)* | (0.490,0.685) | 0.002 (0.001)* | (0.001,0.004) |  | 1 | 0.005 (0.026) |
| Psychological Distress | 0.237 (0.065)* | (0.070,0.405) | 0.711 (0.009)* | (0.688,0.734) |  | 0.226* (0.041) | 1 |
| Ethnicity | -0.104 (0.275) | (-0.813,0.605) | -0.050 (0.026) | (-0.116,0.016) |  | ‒ | ‒ |
| SES | -0.006 (0.003) | (-0.014,0.002) | -0.001 (<0.001)* | (-0.002,<0.001) |  | -0.367 (0.901) | -0.941* (0.115) |
| Gender | -0.539 (0.128)* | (-0.870,-0.209) | -0.003 (0.011) | (-0.032,0.025) |  | ‒ | ‒ |

*Notes. n* = 7,869.

Ethnicity is coded as 1 = NZ Euro, 0 = other. SES = Socio Economic Status. Gender is coded as 0 = Women, 1 = Men.
^#^ modelled as covariances. Covariance above the diagonal is T4 covariance.
**p* < .01

**Life Satisfaction**

**Supplementary Table 7a**

Summary of Intercorrelations, Means, and Standard Deviations for Cross Lagged Panel Model for Times 1, 2, 3, and 4 of Life Satisfaction and Social Media Use for Younger Adults

|  | *M* | *(SD)* | *N* | 1 | 2 | 3 | 4 | 5 | 6 | 7 | 8 | 9 | 10 |
| --- | --- | --- | --- | --- | --- | --- | --- | --- | --- | --- | --- | --- | --- |
| 1. Social Media Use T1 | 6.504 | (9.730) | 1,922 | 1 |  |  |  |  |  |  |  |  |  |
| 2. Social Media Use T2 | 7.263 | (11.184) | 1,487 | .481 | 1 |  |  |  |  |  |  |  |  |
| 3. Social Media Use T3 | 7.031 | (10.109) | 1,229 | .518 | .562 | 1 |  |  |  |  |  |  |  |
| 4. Social Media Use T4 | 6.650 | (9.474) | 1,258 | .461 | .410 | .552 | 1 |  |  |  |  |  |  |
| 5. Life Satisfaction T1 | 5.106 | (1.230) | 1,900 | -.093 | -.060 | -.062 | -.080 | 1 |  |  |  |  |  |
| 6. Life Satisfaction T2 | 5.104 | (1.281) | 1,486 | -.069 | -.060 | -.022 | -.057 | .727 | 1 |  |  |  |  |
| 7. Life Satisfaction T3 | 5.107 | (1.275) | 1,219 | -.093 | -.052 | -.049 | -.040 | .669 | .728 | 1 |  |  |  |
| 8. Life Satisfaction T4 | 5.122 | (1.256) | 1,267 | -.029 | -.064 | -.073 | -.045 | .652 | .683 | .714 | 1 |  |  |
| 9. Gender T1 | .299 | (.457) | 1,943 | -.078 | -.065 | -.118 | -.135 | -.107 | -.113 | -.087 | -.124 | 1 |  |
| 10. Ethnicity T1 | .853 | (.354) | 1,943 | -.059 | -.052 | -.001 | -.048 | .061 | .069 | .050 | .024 | -.032 | 1 |
| 11. SES T1 | 54.817 | (16.651) | 1,927 | -.078 | -.045 | -.051 | -.090 | .170 | .145 | .147 | .182 | -.008 | .009 |

*Notes. n* = 1,943.
Ethnicity is coded as 1 = NZ Euro, 0 = other. SES = Socio Economic Status. Gender is coded as 0 = Women, 1 = Men.

*p* < .01 where *r* ≥ .059.

**Supplementary Table 7b**

Path Coefficients of the Associations Between Social Media Use and Life Satisfaction for Younger Adults.

| Predictors T-1 | Outcome *T* |  |  |  |  | Covariates T1^#^ | |
| --- | --- | --- | --- | --- | --- | --- | --- |
|  | Social Media |  | Life Satisfaction |  |  | Social Media Use | Life Satisfaction |
|  | *B (SE)* | *99% CI* | *B (SE)* | *99% CI* |  |  |  |
| Social Media | 0.521 (0.043)* | (0.410,0.631) | -0.002 (0.001) | (-0.006,0.002) |  | 1 | 0.084 (0.196) |
| Life Satisfaction | -0.106 (0.089) | (-0.336,0.124) | 0.724 (0.016)* | (0.682,0.766) |  | -1.118* (0.366) | 1 |
| Ethnicity | -1.251 (0.760) | (-3.210,0.707) | -0.043 (0.078) | (-0.245,0.158) |  | ‒ | ‒ |
| SES | -0.034 (0.017) | (-0.078,0.010) | 0.005 (0.002)* | (0.001,0.009) |  | -12.799* (3.780) | 3.478* (0.486) |
| Gender | -1.441 (0.395)* | (-2.458,-0.425) | -0.156 (0.056)* | (-0.3005,-0.013) |  | ‒ | ‒ |

*Notes. n* = 1,943.
Ethnicity is coded as 1 = NZ Euro, 0 = other. SES = Socio Economic Status. Gender is coded as 0 = Women, 1 = Men.

^#^ modelled as covariances. Covariance above the diagonal is T4 covariance.
**p* < .01

**Supplementary Table 7c**

Summary of Intercorrelations, Means, and Standard Deviations for Cross Lagged Panel Model for Times 1, 2, 3, and 4 of Life Satisfaction and Social Media Use for Mid Aged Adults

|  | *M* | *(SD)* | *N* | 1 | 2 | 3 | 4 | 5 | 6 | 7 | 8 | 9 | 10 |
| --- | --- | --- | --- | --- | --- | --- | --- | --- | --- | --- | --- | --- | --- |
| 1. Social Media Use T1 | 3.765 | (6.303) | 3,997 | 1 |  |  |  |  |  |  |  |  |  |
| 2. Social Media Use T2 | 4.057 | (6.443) | 3,303 | .477 | 1 |  |  |  |  |  |  |  |  |
| 3. Social Media Use T3 | 3.979 | (6.314) | 2,743 | .504 | .666 | 1 |  |  |  |  |  |  |  |
| 4. Social Media Use T4 | 3.911 | (6.51) | 2,912 | .480 | .521 | .461 | 1 |  |  |  |  |  |  |
| 5. Life Satisfaction T1 | 5.207 | (1.198) | 3,970 | -.054 | -.045 | -.044 | -.044 | 1 |  |  |  |  |  |
| 6. Life Satisfaction T2 | 5.229 | (1.218) | 3,298 | -.080 | -.077 | -.079 | -.072 | .749 | 1 |  |  |  |  |
| 7. Life Satisfaction T3 | 5.237 | (1.235) | 2,711 | -.082 | -.058 | -.049 | -.047 | .717 | .754 | 1 |  |  |  |
| 8. Life Satisfaction T4 | 5.291 | (1.198) | 2,959 | -.082 | -.056 | -.051 | -.050 | .674 | .704 | .761 | 1 |  |  |
| 9. Gender T1 | .329 | (.470) | 4,079 | -.113 | -.107 | -.117 | -.100 | -.066 | -.050 | -.059 | -.089 | 1 |  |
| 10. Ethnicity T1 | .888 | (.315) | 4,079 | -.086 | -.100 | -.114 | -.094 | .034 | .036 | .031 | .062 | -.006 | 1 |
| 11. SES T1 | 56.204 | (16.044) | 4,037 | -.035 | -.042 | -.033 | -.051 | .140 | .124 | .122 | .120 | -.069 | .059 |

*Notes. n* = 4,079.
Ethnicity is coded as 1 = NZ Euro, 0 = other. SES = Socio Economic Status. Gender is coded as 0 = Women, 1 = Men.

*p* < .01 where *r* ≥ .041.

**Supplementary Table 7d**

Path Coefficients of the Associations Between Social Media Use and Life Satisfaction for Mid Aged Adults.

| Predictors T-1 | Outcome *T* |  |  |  |  | Covariates T1^#^ | |
| --- | --- | --- | --- | --- | --- | --- | --- |
|  | Social Media |  | Life Satisfaction | |  | Social Media Use | Life Satisfaction |
|  | *B (SE)* | *99% CI* | *B (SE)* | *99% CI* |  |  |  |
| Social Media | 0.552 (0.040)* | (0.450,0.654) | -0.004 (0.002) | (-0.008,<0.001) |  | 1 | -0.066 (0.082) |
| Life Satisfaction | -0.132 (0.051)* | (-0.264,<0.001) | 0.756 (0.011)* | (0.728,0.784) |  | -0.417* (0.148) | 1 |
| Ethnicity | -0.701 (0.585) | (-2.207,0.805) | 0.147 (0.064) | (-0.018,0.312) |  | ‒ | ‒ |
| SES | -0.012 (0.008) | (-0.032,0.007) | 0.002 (0.001) | (-0.001,0.004) |  | -3.579 (1.767) | 2.682* (0.319) |
| Gender | -0.605 (0.206)* | (-1.136,-0.075) | -0.111 (0.033)* | (-0.195,-0.026) |  | ‒ | ‒ |

*Notes. n* = 4,079.
Ethnicity is coded as 1 = NZ Euro, 0 = other. SES = Socio Economic Status. Gender is coded as 0 = Women, 1 = Men.
^#^ modelled as covariances. Covariance above the diagonal is T4 covariance.
**p* < .01

**Supplementary Table 7e**

Summary of Intercorrelations, Means, and Standard Deviations for Cross Lagged Panel Model for Times 1, 2, 3, and 4 of Life Satisfaction and Social Media Use for Older Aged Adults

|  | *M* | *(SD)* | *N* | 1 | 2 | 3 | 4 | 5 | 6 | 7 | 8 | 9 | 10 |
| --- | --- | --- | --- | --- | --- | --- | --- | --- | --- | --- | --- | --- | --- |
| 1. Social Media Use T1 | 2.359 | (4.989) | 7,609 | 1 |  |  |  |  |  |  |  |  |  |
| 2. Social Media Use T2 | 2.653 | (5.600) | 6,799 | .546 | 1 |  |  |  |  |  |  |  |  |
| 3. Social Media Use T3 | 2.662 | (4.992) | 5,830 | .550 | .596 | 1 |  |  |  |  |  |  |  |
| 4. Social Media Use T4 | 2.550 | (5.425) | 5,990 | .456 | .454 | .585 | 1 |  |  |  |  |  |  |
| 5. Life Satisfaction T1 | 5.276 | (1.161) | 7,617 | -.069 | -.053 | -.061 | -.052 | 1 |  |  |  |  |  |
| 6. Life Satisfaction T2 | 5.295 | (1.147) | 6,852 | -.058 | -.045 | -.054 | -.049 | .742 | 1 |  |  |  |  |
| 7. Life Satisfaction T3 | 5.334 | (1.164) | 5,782 | -.056 | -.051 | -.054 | -.039 | .726 | .755 | 1 |  |  |  |
| 8. Life Satisfaction T4 | 5.405 | (1.127) | 6,124 | -.037 | -.042 | -.032 | -.035 | .703 | .722 | .746 | 1 |  |  |
| 9. Gender T1 | .414 | (.493) | 7,870 | -.109 | -.115 | -.111 | -.105 | -.037 | -.038 | -.022 | -.054 | 1 |  |
| 10. Ethnicity T1 | .912 | (.283) | 7,870 | -.065 | -.064 | -.082 | -.055 | .030 | .046 | .030 | .035 | -.037 | 1 |
| 11. SES T1 | 53.325 | (15.793) | 7,800 | -.004 | .007 | .015 | -.006 | .095 | .080 | .069 | .084 | -.074 | .069 |

*Notes. n* = 7,870.
Ethnicity is coded as 1 = NZ Euro, 0 = other. SES = Socio Economic Status. Gender is coded as 0 = Women, 1 = Men.
*p* < .01 where *r* ≥ .030.

**Supplementary Table 7f**

Path Coefficients of the Associations Between Social Media Use and Life Satisfaction for Older Aged Adults.

| Predictors T-1 | Outcome *T* |  |  |  |  | Covariates T1^#^ |  |
| --- | --- | --- | --- | --- | --- | --- | --- |
|  | Social Media |  | Life Satisfaction | |  | Social Media Use | Life Satisfaction |
|  | *B (SE)* | *99% CI* | *B (SE)* | *99% CI* |  |  |  |
| Social Media | 0.589 (0.038)* | (0.492,0.687) | -0.002 (0.001) | (-0.005,0.001) |  | 1 | -0.061 (0.043) |
| Life Satisfaction | -0.095 (0.030)* | (-0.174,-0.017) | 0.755 (0.007)* | (0.735,0.774) |  | -0.388* (0.082) | 1 |
| Ethnicity | -0.111 (0.277) | (-0.823,0.602) | 0.027 (0.046) | (-0.092,0.145) |  | ‒ | ‒ |
| SES | -0.006 (0.003) | (-0.014,0.002) | 0.002 (0.001)* | (<0.001,0.004) |  | -0.368 (0.901) | 1.740* (0.214) |
| Gender | -0.552 (0.129)* | (-0.884,-0.219) | -0.077 (0.020)* | (-0.129,-0.024) |  | ‒ | ‒ |

*Notes. n* = 7,870.
Ethnicity is coded as 1 = NZ Euro, 0 = other. SES = Socio Economic Status. Gender is coded as 0 = Women, 1 = Men.
^#^ modelled as covariances. Covariance above the diagonal is T4 covariance.
**p* < .01
